# Role of SOX10 Immunohistochemical Expression in Diagnosing Triple Negative Breast Cancer and Its Correlation With Clinicopathological Features

**DOI:** 10.7759/cureus.59276

**Published:** 2024-04-29

**Authors:** Muhammad Usman Tariq, Muhammad Afnan Siddiqui, Nasir Ud Din, Naila Kayani

**Affiliations:** 1 Histopathology, Al Hada Armed Forces Hospital, Taif, SAU; 2 Histopathology, Indus Hospital and Health Network, Karachi, PAK; 3 Pathology and Laboratory Medicine, Aga Khan University Hospital, Karachi, PAK

**Keywords:** tumor infiltrating lymphocytes, immunohistochemistry, tnbc, triple negative breast cancer, sox10

## Abstract

Background: Triple-negative breast cancer (TNBC) poses a diagnostic challenge for histopathologists due to the reduced frequency of breast-specific markers. SOX10 has emerged as a useful diagnostic marker for TNBC. The aim of our study was to determine the frequency of SOX-10 immunohistochemical (IHC) expression in our cohort and assess its correlation with clinicopathological and histological features.

Materials and methods: We included 72 primary TNBC cases. Specimens included tru-cut biopsies and excision specimens. We stained whole slide sections of these specimens with SOX10 antibody and calculated its frequency (%) of expression and H-score. We applied the chi-square test to assess the correlation between SOX10 expression and clinicopathological and histological features such as the patient's age, specimen type, tumor size, histological type, histological grade, nuclear pleomorphism, mitotic count, tumor-infiltrating lymphocytes (TILs), necrosis, calcification, lymphovascular invasion (LVI), lymph node involvement, T stage, and N stage.

Results: SOX10 expression was observed in 42 (58.3%) cases with a median H-score of 57.5. The expression was significantly higher in tru-cut biopsy specimens as compared to excision specimens (73.5 vs 41.7%) and TILs negative tumors as compared to TILs positive tumors (64.3% vs 27.3). Metaplastic carcinoma showed reduced expression when compared with non-metaplastic tumors (35.7% vs 63.8%), but statistical significance was not achieved. No correlation was observed with the patient's age, tumor size, histological type, histological grade, nuclear pleomorphism, mitotic count, necrosis, calcification, LVI, lymph node involvement, T stage, and N stage.

Conclusion: SOX10 was expressed in more than half of the TNBC cases of our study which not only highlights its diagnostic utility but advocated its application in combination with other breast-specific markers. The expression didn’t correlate with the majority of clinicopathological and histological features, but correlation with tru-cut biopsy specimens and absence of TILs draws attention towards possible roles of proper fixation and host immunity, respectively.

## Introduction

Breast cancer (BC) is the most common malignancy worldwide, accounting for 11.7% of all cancers [1]. Developing countries of Asia with historically low incidence rates have seen a rapid rise in BC incidence during the last few decades. Pakistan has the highest age-standardized incidence rate in South Asia and the highest age-standardized mortality rate in Asia [2-4]. Triple-negative breast cancer (TNBC) is a molecularly heterogeneous subgroup of BC that is characterized by negative expression for three biomarkers namely estrogen receptors (ER), progesterone receptors (PR), and human epidermal growth factor receptor-2 (HER-2). TNBC comprises 10-45% of all BC [5-9]. TNBCs are further divided into basal-like BC (BLBC) and non-basal-like BC (NBLBC) [10]. In Asian countries, it presents more commonly in younger age, with larger size and high histological grade [3,4]. TNBC bears a higher risk of visceral metastasis and demonstrates a poor prognosis [11]. Metastatic progression is the most significant cause of mortality in BC patients [8]. In addition, these patients have an increased risk of developing second primary carcinoma of other organs during the first five years of diagnosis [12].

In patients with metastatic disease, optimum treatment and management rely on correct diagnosis, therefore, all surgically accessible metastatic tumors (suspected BC) should be biopsied for confirmation of breast origin and reassessment of biomarkers [8]. Determining the primary tumor in metastatic tumor biopsies is a routine task for histopathologists. Clinical history is of paramount importance and aid but in some tumors either it is not provided with the specimen, metastasis is the initial presentation, or primary BC is occult [13,14]. Due to the diversity and overlapping of morphological features, the distinction is not possible on routine hematoxylin and eosin (H&E) examination. Metastatic BC can morphologically mimic carcinoma of the lung, gynecological, and gastrointestinal tracts [14]. In cases of known primary BC, a re-review of slides for comparison of morphological features of both primary and metastatic tumors is performed, however, immunohistochemical (IHC) markers are routinely applied in all metastatic cases for confirmation of the primary site. Breast-specific IHC markers including GATA3, mammoglobin, and GCDFP15 are generally helpful in overall BC cases but their expression is reduced in TNBC [8,9,15-17]. Moreover, the IHC expressions of these markers are not completely concordant between primary and metastatic tumors of the same case [8,9,13,14,16]. Some of the biomarker-positive primary tumors lose biomarker expression after metastasis with a discordance rate of up to 31% [8]. The sensitivity of these markers improves when applied in combination [15,16].

Different studies have attempted to evaluate the diagnostic utility of various IHC markers (as individual or combination) in primary and metastatic TNBC cases [9,13,14,16,17]. GATA3 is the most sensitive marker for overall BC but its sensitivity decreases in TNBC [10,14]. SOX10 has emerged as a sensitive IHC marker for TNBC which also shows better concordance between primary and metastatic tumors [5,8,9,14,16,18-20]. Furthermore, the most sensitive combination of breast-specific markers comprises SOX10 and GATA3 [7,8,14,19]. Some studies have also identified a correlation between SOX10 and clinicopathological features [9,19,21-23].

In this study, we aimed to determine the frequency of SOX10 expression in TNBC. We also attempted to find a correlation between SOX10 expression and clinicopathological features.

## Materials and methods

This study was approved by the institutional ethics review committee of the Aga Khan University Hospital (2020-5179-14268). The surgical pathology database was searched for primary TNBC cases diagnosed between 2019 and 2022 at the histopathology section of the Department of Pathology and Laboratory Medicine, Aga Khan University Hospital. Cases in which formalin fixed paraffin embedded tissue blocks were not available, poorly fixed specimens, and post neo-adjuvant specimen were excluded. A total of 72 primary TNBC cases were included in the study. Clinicopathological data regarding the patient's age and gender was collected from the patient’s medical records. Histological features including specimen type, histological type, histological grade, nuclear pleomorphism, mitotic count, necrosis, calcification, lymphovascular invasion (LVI), tumor-infiltrating lymphocytes (TILs), tumor size, tumor stage (T stage), lymph node status and nodal stage (N stage) were recorded from pathology reports.

IHC staining of paraffin-embedded tissue sections was carried out using the Dako EnVision System (Agilent Technologies, Santa Clara, CA), following the manufacturer's protocols. Four-micron thick sections were taken from formalin-fixed paraffin-embedded tissue blocks and placed on glass slides. High pH buffer Dako PT Link (Agilent Technologies, Santa Clara, CA) was used for deparaffinization, rehydration, and epitope retrieval in the pretreatment process. EnVision FLEX peroxidase-blocking reagent (Agilent Technologies, Santa Clara, CA) was applied first on the slides for five minutes and then the slides were washed with a wash buffer. The primary antibody used was an anti-SOX10 mouse monoclonal antibody (Clone 55k-2, diluted at 1:100, Santa Cruz Biotechnology, Santa Cruz, CA). Primary antibody was applied on slides, incubated for 20 minutes, and then washed with wash buffer. EnVision FLEX/HRP (secondary antibody) was then applied, incubated for 20 minutes, and subsequently washed. EnVision FLEX DAB (diaminobenzidine)+chromogen diluted in EnVision FLEX substrate buffer was then applied for five minutes and subsequently washed. Counter-staining was done with hematoxylin. Finally, the slide was dehydrated (graduated alcohol to xylene). Lastly, a slide was mounted with DPX (dibutylphthalate polystyrene xylene) and a cover slip was applied. Known positive cases of malignant melanoma or peripheral nerve sheath tumors were used as positive external control and tonsil tissue as negative external control which were run simultaneously using the same protocol.

The slides were then microscopically examined by two pathologists for estimation of SOX10 expression in tumor cells. IHC expression of SOX-10 was assessed in the nucleus of tumor cells. The H-score was used for the interpretation of IHC expression of SOX10 which was obtained by multiplying the percentage of positive tumor cells by the staining intensity of tumor cell nuclei (0; no staining, 1; weak, 2; moderate, and 3; strong). SOX10 expression was considered positive if the H-score was ≥10 [19].

Statistical analysis was performed using the statistical software Statistical Package for Social Sciences (SPSS), version 22.0 (IBM Corp., Armonk, NY). A descriptive statistical analysis of quantitative/continuous and qualitative/categorical data was performed. Mean and standard deviation were calculated for the patient's age and tumor size. Frequency and percentages will be calculated for age groups, specimen type, histological type, histological grade, nuclear pleomorphism, mitotic count, necrosis, calcification, LVI, TILs, tumor size, T stage, lymph node status and N stage and SOX-10 expression. Pearson’s chi-square test or Fisher’s exact test was used to examine the association between SOX10 expression and clinicopathological and histological features. A p-value of ≤ 0.05 was considered statistically significant.

## Results

The clinicopathological features of the patients are summarized in Table 1. The patient’s age ranged from 23-80 years. The median age was 45 years, and the mean ± SD was 45.2 ± 12.6. The majority of the patients aged between 40 and 60 years. Almost half of the specimens were tru-cut biopsies. Invasive carcinoma of no special type (ICNST) was the most common histologic type of breast carcinoma followed by metaplastic carcinoma. More than 80% of the tumors were histological grade III. In excision specimens, tumor size ranged from 1.1-12.5 cm. The median size was 3.5 cm and the mean ± SD was 4.3 ± 2.4. The majority of the tumors that underwent excision and lymph node excision, qualified for T stage T2 and N stage N0, respectively (Table 1).

**Table 1:** Summary of clinicopathological and histological features of triple-negative breast cancer patients (n=72). ICNST: Invasive carcinoma of no special type; IHC: Immunohistochemical. *These clinicopathological features have been assessed in excisional biopsy specimens. ** N stage has been assessed in excisional biopsy cases with lymph node excision.

Clinicopathological and histological features	Expression
Patient’s age groups	
< 40 years	28 (38.9%)
40-60 years	35 (48.6%)
> 60 years	9 (12.5%)
Specimen type	
Tru-cut biopsy	34 (47.2%)
Outside blocks of review	2 (2.8%)
Excision	50%)
Mastectomy	21(29.2%)
Wide local excision/Lumpectomy	15 (20.8%)
Lymph node excision	24 (33.3%)
Histologic type of invasive carcinoma	
Invasive carcinoma of no special type (ICNST)	55 (76.4%)
Metaplastic carcinoma	14 (19.4%)
ICNST with micropapillary component	2 (2.8%)
ICNST with solid papillary component	1 (1.4%)
Histologic grade of invasive carcinoma	
Grade I	0
Grade II	13 (18.1%)
Grade III	59 (81.9%)
Nuclear pleomorphism	
Mild	2 (2.8%)
Moderate	15 (20.8%)
Marked	55 (76.4%)
Mitotic count per 10 high-power fields	
≤ 7 mitoses	4 (5.6%)
8-14 mitoses	29 (40.2%)
≥15 mitoses	39 (54.2%)
Necrosis (n=36)*	20 (55.6%)
Lymphovascular invasion (n=36)*	9 (25%)
Tumor-infiltrating lymphocytes (n=36)*	22 (61.1%)
Calcification (n=36)*	5 (13.9%)
Lymphovascular invasion (n=36)*	1 (2.8%)
T stage (n=36)*	
T1	4 (11.1%)
T2	19 (52.8%)
T3	13 (36.1%)
N stage (n=24)**	
N0	10 (41.6%)
N1	5 (20.8%)
N2	5 (20.8%)
N3	4 (16.7%)
Cumulative H-score of SOX10 IHC stain	
Range	0-290
Mean ± SD	83.4 ± 90.8

**Figure 1:**
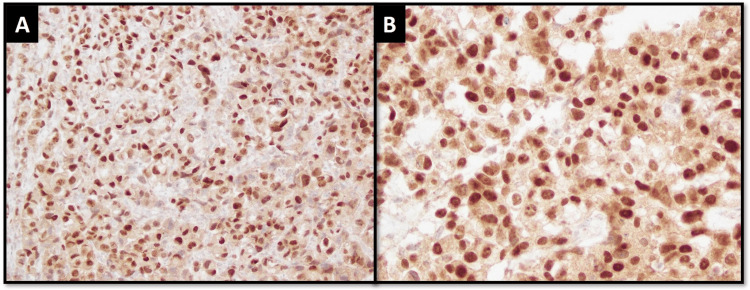
SOX10 IHC expression. (A) Intermediate view and (B) high-power view of tumor cells demonstrating moderate to strong nuclear expression for SOX10 IHC stain. IHC: Immunohistochemical.

Positive SOX10 expression was observed in 42 (58.3%) cases (Figure 1). The median cumulative H-score was 57.5.

The clinicopathological and histological features were correlated with SOX10 expression and summarized in Table 2. The proportion of positive SOX10 expression was significantly higher in tru-cut biopsies and in tumors with the absence of tumor-infiltrating lymphocytes. Reduced expression of SOX10 stain was observed in metaplastic carcinoma when compared with other histologic types (35.7% vs 63.8%), but this difference was not statistically significant (Table 2).

**Table 2:** Comparison of clinicopathological and histological features with SOX10 IHC expression (n=72). ICNST: Invasive carcinoma of no special type; IHC: Immunohistochemical. *Outside cases received for review are excluded. **These clinicopathological features have been assessed in excisional biopsy specimens. *** N stage has been assessed in excisional biopsy cases with lymph node excision.

Clinicopathological and histological features	SOX10 positive	SOX10 negative	p-value
Patient’s age groups			p=0.104
< 40 years	20 (71.4%)	8 (28.6%)	
40-60 years	19 (54.3%)	16 (45.7%)	
>60 years	3 (33.3%)	6 (66.7%)	
Specimen type (n=70)*			p=0.007
Tru-cut biopsy	25 (73.5%)	9 (26.5%)	
Excision specimen	15 (41.7%)	21 (58.3%)	
Histologic type of invasive carcinoma			p=0.226
Invasive carcinoma of no special type (ICNST)	35 (63.6%)	20 (36.4%)	
Metaplastic carcinoma	5 (35.7%)	9 (64.3%)	
ICNST with micropapillary component	1 (50%)	1 (50%)	
ICNST with solid papillary pattern	1 (100%)	-	
Histologic type of invasive carcinoma			p=0.056
Metaplastic carcinoma	5 (35.7%)	9 (64.3%)	
Non-metaplastic carcinoma (ICNST and other types)	37 (63.8%)	21 (36.2%)	
Histologic grade of invasive carcinoma			p=0.379
Grade II	9 (69.2%)	4 (30.8%)	
Grade III	33 (55.9%)	26 (44.1%)	
Nuclear pleomorphism			p=0.160
Mild	1 (50%)	1 (50%)	
Moderate	12 (80%)	3 (20%)	
Marked	29 (52.7%)	26 (47.3%)	
Mitotic count per 10 high-power fields			p=0.844
≤ 7 mitoses	2 (50%)	2 (50%)	
8-14 mitoses	18 (62.1%)	11 (37.9%)	
≥15 mitoses	22 (56.4%)	17 (43.6%)	
Necrosis (n=36)**			p=0.364
Positive	7 (35%)	13 (65%)	
Negative	8 (50%)	8 (50%)	
Lymphovascular invasion (n=36)**			p=1.000
Positive	4 (44.4%)	5 (55.6%)	
Negative	11 (40.7%)	16 (59.3%)	
Tumor-infiltrating lymphocytes (n=36)**			p=0.028
Present	6 (27.3%)	16 (72.7%)	
Absent	9 (64.3%)	5 (35.7%)	
T stage (n=36)**			p=0.817
T1	2 (50%)	2 (50%)	
T2	7 (36.8%)	12 (63.2%)	
T3	6 (46.2%)	7 (53.8%)	
Lymph node involvement (n=28)			p=1.000
Present	6 (33.3%)	12 (66.7%)	
Absent	4 (40%)	6 (60%)	
N stage (n=24)***			p=0.400
N0	4 (40%)	6 (60%)	
N1	-	5 (100%)	
N2	2 (40%)	3 (60%)	
N3	1 (25%)	3 (75%)	

A greater proportion of SOX10 positive expression was observed in patients younger than 40 years, tumors having histologic grade II, moderate nuclear pleomorphism, and absence of necrosis, however, statistical significance was not observed (Table 2).

## Discussion

SOX10 was first described in 2008 as a pan-schwannian and melanocytic marker when its expression was reported in all cases of neurofibroma, schwannoma, myoepithelioma of salivary gland, and 97% cases of melanoma [24]. Further studies also observed SOX10 expression in other tumors including clear cell sarcoma, granular cell tumors, salivary gland tumors with myoepithelial differentiation, gastrointestinal stromal tumors, and gliomas [14,25-28]. Among carcinoma, SOX10 expression has been most frequently observed in salivary gland carcinoma and breast carcinoma, along with rare expression in head and neck squamous cell carcinoma, clear cell carcinoma, lung adenocarcinoma (LA), urothelial carcinoma, and colorectal carcinoma [14,25]. SOX10 is not only expressed in myoepithelial cells but also in some luminal cells of benign breast acini which serve as its internal control [7,13,20,25].

The frequency of SOX10 expression in overall BC has ranged from 6.5-40% [5,7,9,14,20,25], while the frequency of expression in primary TNBC has ranged from 25-87.5% [5,7-9,13,14,16,17,19,20-23,29]. We also observed SOX10 expression in 58.3% of cases in our cohort. The majority of the studies have reported moderate to strong staining in at least 40% of tumor cells in most cases of their cohorts [5,7,9,13,14,17,20,29]. The cutoff for labeling positivity used in different studies has been nuclear staining in ≥1% of tumor cells, irrespective of staining intensity [5,7-9,13,19,20,22,29]. A single study used the cutoff of >10% positive tumor cells [17]. In a study evaluating the H-score for SOX10 expression, the median H-score was 80 [14]. We also evaluated the H-score in our study and observed a median H-score of 57.5. In some studies, SOX10 expression was heterogeneous but easily recognizable due to presence of areas showing diffuse expression and moderate to strong staining [5,7,20]. We also observed heterogeneous intensity SOX10 staining in tumor cells. The expression was randomly distributed across the tumor and no specific staining pattern was observed with respect to the tumor center or periphery.

Different studies have compared SOX10 expression among different subtypes of BC using different study designs and methodologies and the expression was higher in TNBC and ER-negative (ER-) subtypes as compared to ER-positive (ER+) and HER-2 positive (HER-2+) subtypes [5,7,9,13,14,20,21,29] (summarized in Table 3).

**Table 3:** Summary of findings of other studies evaluating SOX10 expression in TNBC. AR: Androgen receptor; BC: Breast cancer; EGFR: Epidermal growth factor receptor; ER-: Estrogen receptor negative; ER+: Estrogen receptor positive; FOXA1: Forkhead box protein A1; GATA3: GATA binding protein 3; GCDFP15: Gross cystic disease fluid protein 15; IHC: Immunohistochemical; TNBC: Triple-negative breast cancer; TRPS1: Trichorhinophalangeal syndrome type 1; WT1: Wilms' tumor 1. *Primary tumors of metastatic tumors showing triple negative biomarker expression were included. Thirty-nine primary tumors were TNBC and 18 were non-TNBC. The percentage of expression of IHC markers in primary TNBC and non-TNBC was not separately mentioned. **Archived samples (tissue microarray) consisted of primary, metastatic tumors and cell lines. The breakup of the sample tested for SOX10 and p16 was not mentioned. ***Separate frequencies of primary and metastatic tumors were not mentioned.

Authors	Sample size of TNBC cases	Tumor site	Specimen type	Frequency of SOX10 expression in TNBC	Percentages of SOX10 expression in other molecular subtypes of BC	Frequency of other IHC markers’ expression	Correlation with clinicopathological and prognostic features
Nelson et al. [13]	Primary: 8 Metastatic: 8	Paired primary and 1^st^ metastatic site tumor	Tissue microarray	Primary: 2 (25%) Metastatic: 3 (38%)	ER - BC: 71% ER + BC: 0%	GATA3 (n=6): 4 (67%)	No correlation with histologic grade and site of metastatic disease
Aphivatanasiri et al. [14]	Primary: 264 Nodal metastasis: 62	Primary tumors and nodal metastasis	Tissue microarray	Primary: 83 (31.4%) Nodal metastasis: 18 (29%)	Luminal: 2.2% Her2+: 1.3%	Primary: GATA3: 90/263 (34.2%) Mammoglobin 74/264 (28%) GCDFP15: 69/269 (25.7%) Nodal metastasis: GATA3: 20/72 (27.8%) Mammoglobin: 9/45 (20%) GCDFP15: 14/41 (34.1%)	Not performed
Laurent et al. [16]	Primary: 207 Intrathoracic metastasis: 18	Primary tumors and intrathoracic metastasis	Tissue microarray	Primary: 129 (62.3%) Intrathoracic metastasis: 9 (50%)		Primary (n=207): Mammoglobin: 79 (38.2%) GATA3: 63 (30.4%) AR: 62 (30%) GCDFP15: 43 (20.8%) Intrathoracic metastasis (n=18): Mammoglobin: 10 (55.5%) GCDFP15: 7 (38.9%) GATA3: 6 (33.3%) AR: 6 (33.3%)	Not performed
Harbhajanka et al. [19]	48	Primary tumors	Tissue microarray	18 (37.5%)	Not performed	AR: 16 (33.3%) WT1: 11 (23.4%) GATA3: 32 (66.7%) GCDFP15: 11 (23.9%) Mammoglobin: 17 (36.2%)	No correlation with age, tumor grade, stage, lymph node involvement and recurrence. Positive correlation with WT1 and negative correlation with AR expressions
Jamidi et al. [7]	Test cohort: 265 Validation cohort: 42	Primary tumors	Test cohort: Tissue microarray Validation cohort: Whole sections	Test cohort: 83 (31.3%) Validation cohort: 34 (81%)	Test cohort: Non-TNBC: 2.6% Validation cohort: Non-TNBC: 2%	Test cohort: GATA3: 91/264 (34.5%) Mammoglobin 74/265 (27.9%) GCDFP15: 69/274 (25.2%) Validation cohort: GATA3: 17/42 (40.4%) Mammoglobin: 23/42 (54.8%) GCDFP15: 9/42 (21.4%)	Positive correlation with younger age, higher histologic grade, necrosis, high Ki-67 index and basal markers. Negative correlation with apocrine features and negative AR expression
Cimino-Mathews et al. [5]	45	Primary tumors	Tissue microarray	32 (71.1%)	ER+ or Her2+ subgroup: 5%	Not performed	Not performed
Tozbikian et al. [8]	Primary tumors: 39 Metastatic tumors: 57	Metastatic TNBC with matched primary tumors*	Whole sections	Metastatic tumors: 33 (58%)	Not performed	Metastatic tumors: GATA3: 47 (82%) AR: 14 (25%)	Not performed
Chiu et al. [20]	10	Primary tumors	Whole sections	6 (60%)	Non-TNBC: 3%	Not performed	Not performed
Qazi et al. [9]	38	Primary tumors	Tissue microarray	28 (74%)	ER+: 3% (50% ER-low, 20% ER-intermediate and 0% ER-high subgroups) ER-: 64% Her2+: 33.3%	GATA3: 24 (63%)	Not performed
Yoon et al. [29]	Samples with clinical data: 56 Archived samples: 147**	Samples with clinical data: Primary tumors	Tissue microarray	Samples with clinical data: 48 (85.7%) Archived samples: 117 (80%)	Non-TNBC: 35%	Samples with clinical data: P16: 44 (78.6%) AR: 15 (26.8%) CK5/6: 24 (42.9%) Archived samples: P16: 91/163 (56%) AR & CK5/6: Not tested	No correlation with survival
Jin et al. [21]	Primary tumors: 71 Metastatic tumors: 22	Primary and metastatic tumors	Whole sections	Primary tumors: 48 (67.6%) Metastatic tumors: 22 (68.2%)	Primary tumors: Luminal (A,B): 0% Her2: 3.1% Metastatic tumors: Luminal (A,B): 0% Her2: 9.1%	Primary tumors: GATA3: 62 (87.3%) GCDFP15: 7 (9.9%) Mammoglobin: 14 (19.7%) FOXA1: 21 (29.6%) Metastatic tumors: GATA3: 4 (18.2%) GCDFP15: 3 (13.6%) Mammoglobin: 5 (22.7%) FOXA1: 21 (29.6%)	Positive correlation with histological grade III, clinical stage III and N stage N2. Negative correlation with disease-free survival.
Ali et al. [22]	100	Primary tumors	Tissue microarray	67 (67%)	Not performed	Not performed	Positive correlation with histological grade III. No correlation with age, size and stage.
Kriegsmann et al. [23]	113	Primary tumors	Tissue microarray	46 (41%)	Not performed	P53: 56 (50%) Vimentin: 44 (39%) EGFR: 40 (35%) CD117: 18 (16%) AR: 8 (7%)	Positive correlation with T stage T1, CD117 and vimentin expression. No significant difference in survival.
Yoon et al. [17]	292 Primary tumors: 246 Metastatic tumors: 46	Primary & metastatic tumors	Whole sections	Overall frequency***: 167 (57.2%)	Not performed	Overall frequency***: TRPS1: 284 (97.3%) GATA3: 172 (58.9%)	Not performed
Our study	72	Primary tumors	Whole sections	42 (58.3%)	Not performed	Not performed	Positive correlation with tru-cut biopsy specimen type. Negative correlation with tumor-infiltrating lymphocytes.

In a study of 57 metastatic TNBC, Tozbikian et al. observed higher SOX10 expression in metastatic tumors when primary tumors were TNBC (67%) as compared to non-TNBC primary tumors (39%) [8]. Keeping in view the expression of SOX10 in normal myoepithelial cells of breast and salivary gland tumors with myoepithelial differentiation, the higher expression of SOX10 in TNBC and ER- tumors can be explained by their basal-like or myoepithelial differentiation [13].

SOX10 expression has also been studied in different TNBC subgroups. When TNBC was further subdivided into basal-like BC (BLBC) and unclassified subgroups, SOX10 expression was higher in the BLBC subgroup (in primary tumors: 42.2% vs 23% and in nodal metastasis: 42.3% vs 27.3%) [14]. Similarly, Jamidi et al. also observed significantly higher SOX10 expression in the BLBC subgroup as compared to the 5NP subgroup (42.2 vs 22.8%) [7]. In contrast, Cimino-Mathews et al. observed higher expression in the unclassified subgroup as compared to the BLBC subgroup (77% vs 69%) [5]. The authors suggested that the unclassified group might also possess myoepithelial differentiation which is not manifested by IHC markers of basal-like phenotype [5]. Yoon et al. also observed higher expression in non-BLBC as compared to the BLBC subgroup (90.6 vs 79.2%) but this difference was statistically insignificant [29]. Harbhajanka et al. evaluated SOX10 expression in all molecular subtypes of TNBC and reported positivity in 62.5% cases of unstable molecular subtype, 60% basal-like 1, 25% mesenchymal stem cell, 20% basal-like 2, 20% mesenchymal, 20% luminal androgen receptor and 16.7% immunomodulatory subtypes [19]. 

Few studies also compared SOX10 expression in non-metaplastic and metaplastic subgroups of TNBC. A higher frequency of expression was observed in the non-metaplastic as compared to the metaplastic subgroup [5,17]. Similarly, we also observed a higher frequency of SOX10 expression in non-metaplastic carcinoma (63.8%) as compared to metaplastic carcinoma (35.7%).

SOX10 is not only useful for confirming breast origin of tumors at metastatic sites but it is also performed on the breast samples as part of the diagnostic workup for poorly differentiated malignant neoplasm [13]. It can also be helpful in distinguishing metaplastic carcinoma from phyllodes tumor. In Cimino-Mathews et al.’s study SOX10 was positive in 46% of metaplastic carcinoma while it was negative in all 34 phyllodes tumors [5].

Most of the studies on SOX10 have been performed using tissue microarray (TMA) and only a few have used whole sections [7,8,17,20,21]. In Jamidi et al.’s study where test and validation cohorts were evaluated using TMA and whole sections, respectively, the frequency of SOX10 expression was higher in whole sections which led the authors to suggest that the expression can be affected by the amount of tissue [7]. However, we didn’t observe any difference in expression among frequencies reported by different studies using TMA and whole sections (Table 3). In addition, we observed a significantly higher frequency of expression in tru-cut biopsies as compared to excision specimens which also contradicts the view that expression is affected by the amount of tissue. We think that the higher frequency of expression in tru-cut biopsies might be related to special care given to the proper fixation of these samples while larger specimens might face fixation issues related to delay in transportation from different parts of our country.

All breast-specific markers including SOX10, GATA3, mammoglobin, and GCDFP15, expressing positively at the primary tumor site, may lose their expression at the metastatic site and vice versa [13,14]. In overall BC, SOX10 demonstrates the most concordant expression between primary and metastatic tumors (96.4%, κ=0.663) when compared with other breast-specific markers [14]. The frequency of SOX10 expression in metastatic TNBC has ranged from 29-68.2% [8,13,14,16,21]. In TNBC cases, the combination of SOX10 and GATA3 shows the highest sensitivity 60-95% among all dual combinations breast markers [7,8,14]. The combination of SOX10 with GCDFP15 and mammoglobin yields sensitivities of 57.9% and 54%, respectively while the sensitivity of the combination of all four breast markers including SOX10, GATA3, GCDFP15, and mammoglobin rises to 80.2% [7]. Some studies have also reported an inverse correlation between SOX10 and other breast markers including AR, GATA3, mammoglobin, and GCDFP15 [7,8,14,16,19].

The expressions of GATA3 and SOX10 are not completely concordant and independent of each other since GATA3 is considered a luminal marker while SOX10 is considered a basal/myoepithelial marker [7,8,13,17]. In Yoon et al.’s study (including both primary and metastatic BC), 36.6% of cases were SOX10 positive and GATA3 negative while 26.7% cases were GATA3 positive and SOX10 negative [17]. In Laurent et al.’s study, 9/18 metastatic TNBC cases were positive for SOX10, however, it was the only positive breast-specific marker in 4 cases [16].

The main differentials of metastatic tumors in axillary and cervical lymph nodes are BC and LA. The differential expression of SOX10, GATA3, and TTF-1 is usually helpful in determining the tumor origin. However, one should be aware of the rare expression of TTF-1 in BC and SOX10 or GATA3 expression in LA cases [16,30]. Laurent et al. observed the sensitivity and specificity of various breast-specific markers in TNBC and TTF-1 positive and TTF-1 negative LA cohorts. SOX10 was found to be the most sensitive (62.3%) and the most specific (100%) marker [16]. When the combination of SOX10, GATA3, and GCDFP15 was analyzed in TNBC and TTF-1 negative LA cohorts, the sensitivity was raised to 88.4% and specificity to 95.2%. The authors suggested sequential application of SOX10, GATA3, and GCDFP15 for differentiating between TNBC and LA [16].

Another important diagnostic challenge is faced while examining axillary tumors of unknown primary origin which are cytokeratin negative, S100 positive and SOX10 positive. These tumors are considered metastatic melanoma. This IHC profile can also be observed in metaplastic BC cases. In such cases, additional IHC markers should be avoided for differentiating metaplastic BC and melanoma, such as high molecular weight keratins, p63, HMB45, MelanA, etc. [5,20].

Very few studies have correlated SOX10 expression with clinicopathological features and observed significant correlation (Table 3) [7,13,19,21,30]. Positive correlation has been reported with younger age, histological grade III, clinical stage III, pT1 stage, N stage N2, necrosis, high Ki-67 index (≥20%), and basal markers’ expression [21-23], while negative correlation has been reported with apocrine features, negative AR expression and disease-free survival [7,21]. Kriegsmann et al. assessed but couldn’t find any correlation with lymphocytic stroma [23]. In our study, we identified significantly reduced SOX10 expression in tumors with the presence of TILs. This finding would raise the possibility of any association between SOX10 and host immunity.

Limitations

The limitation of our study was a relatively smaller sample which affected the assessment of correlation between SOX10 expression and clinicopathological and histological features. In addition, our study was limited to primary tumor samples and we didn’t assess the diagnostic utility of SOX10 IHC stain in metastatic tumor samples which are more challenging cases in real practice.

## Conclusions

SOX10 is a fairly sensitive marker for triple-negative breast cancer but it should always be used in conjunction with GATA3 immunohistochemical stain. The positive correlation of SOX10 immunohistochemical expression with tru-cut biopsy samples and the negative correlation with tumor-infiltrating lymphocytes point towards possible roles of proper tissue fixation and host immunity in its expression.
